# AI literacy and AI anxiety in nursing students: the serial mediating roles of attitudes and self-efficacy

**DOI:** 10.3389/fpubh.2026.1918134

**Published:** 2026-07-30

**Authors:** Qin Zeng, Shenghua Zhang, Jiacheng Hu, Yajun Wu, Min Yang, Yuan Hu

**Affiliations:** 1 Department of Pediatric Gastroenterology Nursing, West China Second University Hospital, Sichuan University/West China School of Nursing, Sichuan University, Chengdu, Sichuan, China; 2 Key Laboratory of Birth Defects and Related Diseases of Women and Children (Sichuan University), Ministry of Education, Chengdu, Sichuan, China; 3 Department of Pediatric Urinary Disease Center Nursing, West China Second University Hospital, Sichuan University/West China School of Nursing, Sichuan University, Chengdu, Sichuan, China; 4 West China Hospital, Sichuan University/West China School of Nursing, Sichuan, China; 5 Department of Nursing, West China Second University Hospital, Sichuan University/West China School of Nursing, Sichuan University, Chengdu, Sichuan, China; 6 Department of Pediatric Cardiology Nursing, West China Second University Hospital, Sichuan University/West China School of Nursing, Sichuan University, Chengdu, Sichuan, China

**Keywords:** AI anxiety, artificial intelligence literacy, attitudes toward AI, digital public health, health professions education, nursing students, self-efficacy

## Abstract

**Background:**

The rapid integration of artificial intelligence (AI) into health care demands that future nurses develop foundational AI competencies. However, the mechanisms through which AI literacy alleviates AI-related anxiety among nursing students remain inadequately understood, particularly regarding the sequential psychological processes involved.

**Objective:**

This study aimed to test whether attitudes toward AI and AI self-efficacy mediate the relationship between AI literacy and AI anxiety in nursing students, and to examine the serial mediation pathway among these variables.

**Methods:**

A multicenter cross-sectional survey was conducted among 1,482 nursing students from 11 universities in Sichuan Province, China, between May and October 2025. Validated Chinese versions of the Artificial Intelligence Literacy Scale (AILS), General Attitudes toward Artificial Intelligence Scale (GAAIS), Artificial Intelligence Self-Efficacy Scale (AISES), and Artificial Intelligence Anxiety Scale (AIAS) were administered. Structural equation modeling (SEM) was employed to examine direct and indirect effects.

**Results:**

AI literacy was positively correlated with favorable AI attitudes (*r* = 0.285) and AI self-efficacy (*r* = 0.509), and negatively correlated with AI anxiety (*r* = −0.434). The SEM showed a significant negative direct association between AI literacy and AI anxiety (β = −0.329, *P* < 0.001). Significant indirect associations were observed through AI attitudes and AI self-efficacy separately and through the serial pathway AI literacy → attitudes → self-efficacy → anxiety. These findings describe model-based associations and should not be interpreted as evidence of temporal or causal ordering.

**Conclusion:**

Higher AI literacy was associated with lower AI anxiety, and this association was partly accounted for by AI attitudes and AI self-efficacy in the proposed serial mediation model. The observed ordering suggests that more favorable attitudes may be linked to stronger self-efficacy, which may in turn be related to lower anxiety; however, the cross-sectional design does not establish causality. Nursing education may therefore consider combining AI knowledge and skills training with opportunities to develop balanced attitudes and confidence in using AI.

## 1 Introduction

Artificial intelligence (AI) encompasses technologies designed to simulate human cognitive processes, including reasoning, communication, learning and decision-making. The application of AI can enhance nursing students' learning efficiency and facilitate the development of clinical skills, communication, critical thinking and decision-making (1).

However, the rapid expansion of AI technologies has also generated concerns about potential negative impacts. Perceived threats such as job displacement, diminished professional value, reduced self-esteem, increased learning demands, and changes in work patterns may all contribute to individuals' anxiety regarding AI (2, 3). AI anxiety refers to the apprehension or fear arising when individuals perceive that AI technologies might operate beyond their control (4).

Previous studies indicate moderate levels of AI anxiety among nursing students in Korea and Turkey (5–7), while higher levels have been observed in Palestine (8). Factors associated with elevated AI anxiety include younger age, limited AI experience, superficial understanding, restricted access to the internet or smartphones, low trust in AI, and negative perceptions (6–9). Evidence regarding the role of gender is inconsistent (8, 9). Higher AI anxiety may hinder collaboration with AI, inhibit innovation (2), reduce acceptance of AI technologies (10), and negatively affect adoption and sustained use (5, 11). With the growing presence of AI in healthcare, understanding and addressing AI anxiety among nursing students is essential.

AI literacy refers to the ability to identify, use and evaluate AI products while adhering to ethical principles (12). Nursing students with higher AI literacy can critically apply AI to real-world problems (13). Evidence suggests that students with higher levels of AI literacy tend to demonstrate greater interest in AI (14), devote more time and effort to learning AI-related knowledge (15), and exhibit stronger intentions to use AI technologies (16). In addition, nursing students with stronger AI literacy are generally more receptive to emerging technologies in healthcare, exhibit greater innovativeness, and report higher professional self-efficacy (17). A study in Italy found that AI literacy was associated with greater confidence and understanding of technology relevance and with lower anxiety during AI use (18). However, empirical evidence examining this relationship among nursing students remains limited. Therefore, this study proposed research hypothesis 1: AI literacy is negatively associated with AI anxiety among nursing students.

Nursing students' attitudes toward AI vary across countries. Studies in the Philippines and Croatia indicate that most nursing students hold positive views regarding application of AI in nursing practice (19, 20). Similarly, a survey at several universities in western China reported that 81.09% of nursing students expressed positive attitudes toward AI (21). In contrast, a study across ten Middle Eastern countries found that students generally held neutral attitudes, with only about one-third expressing positive perceptions (22). Positive attitudes often stem from recognition of AI's potential benefits, such as improving efficiency, operational stability, and expanding research and clinical opportunities (20, 23). Concerns regarding legal, ethical, privacy, empathy, and employment issues may contribute to negative attitudes (7). Attitudes significantly influence students' willingness to adopt AI (19, 22), and higher AI literacy is linked to more favorable attitudes (13, 24). Positive attitudes may also reduce AI anxiety (5, 25, 26). Therefore, this study proposed research hypothesis 2: Attitudes toward AI mediate the relationship between AI literacy and AI anxiety.

AI self-efficacy refers to individuals' confidence in their ability to effectively use and interact with AI technologies (27). Students with higher self-efficacy are more likely to view AI innovations as opportunities rather than threats (28). They demonstrate greater confidence in academic tasks, initiative in learning, adaptability, self-regulation, and problem-solving skills (29).

AI self-efficacy influences trust, acceptance, and intention to use AI (30, 31). Higher AI literacy is associated with stronger self-efficacy (15), which in turn reduces AI anxiety (32). Therefore, this study proposed research hypothesis 3: AI self-efficacy mediates the relationship between AI literacy and AI anxiety.

The Theory of Reasoned Action (TRA) proposes that individuals' beliefs about a behavior inform their attitudes, which subsequently shape behavioral intentions (33). Applied to the present model, AI literacy may provide the knowledge base through which students evaluate the usefulness, limitations, and risks of AI. Attitude is therefore positioned as the first mediator because it represents an early evaluative response to what students know and believe about AI. More favorable attitudes may be associated with greater willingness to engage with AI tools and learning activities. Such engagement and related mastery experiences may then be associated with stronger AI self-efficacy, reflecting greater confidence in one's ability to use AI (31, 32). Self-efficacy is positioned closer to AI anxiety because perceived capability and control are psychologically relevant to uncertainty and apprehension when interacting with technology. Based on this reasoning, this study proposed research hypothesis 4: AI literacy is associated with AI anxiety through a sequential pathway involving attitudes toward AI and AI self-efficacy.

**Figure 1:**
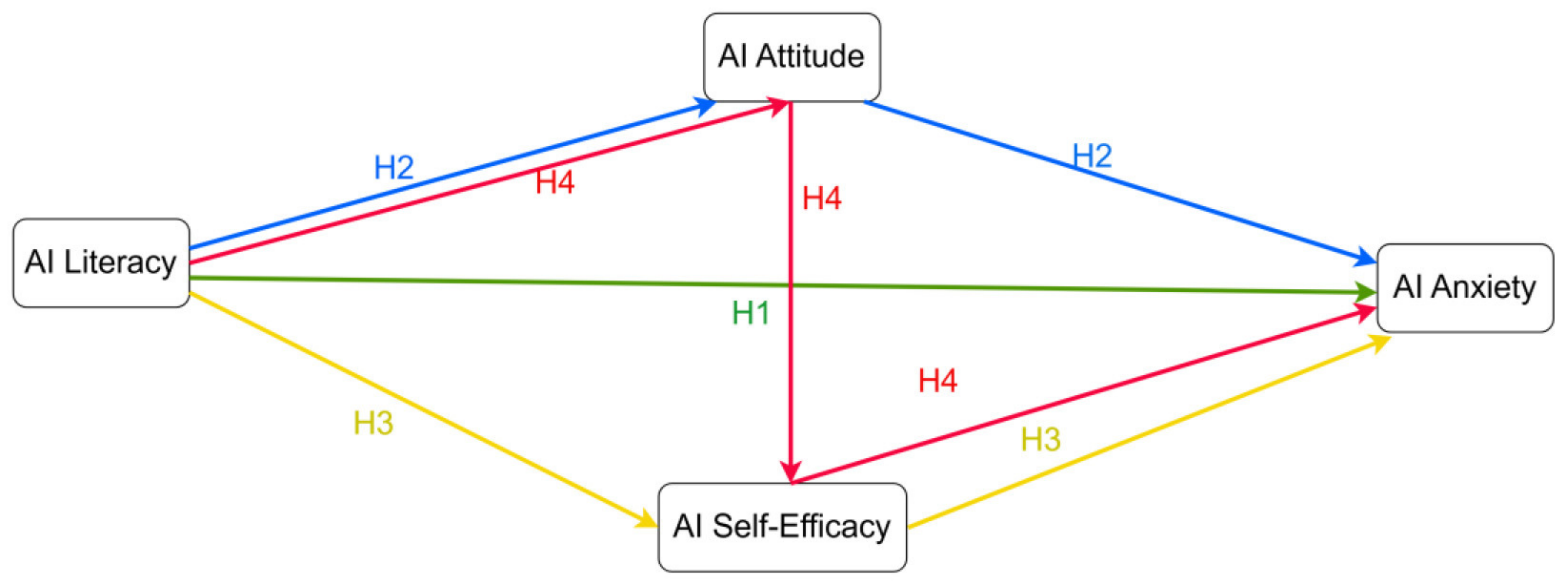
Hypothesized structural model linking AI literacy, AI attitude, AI self-efficacy, and AI anxiety. The model proposes four hypothesized associations: (H1) a direct negative association between AI literacy and AI anxiety; (H2) an indirect association via AI attitude; (H3) an indirect association via AI self-efficacy; and (H4) a serial indirect association in which AI literacy is positively associated with AI attitude, AI attitude is positively associated with AI self-efficacy, and AI self-efficacy is negatively associated with AI anxiety.

This study aimed to examine the relationship between AI literacy and AI anxiety among nursing students, as well as the mediating roles of attitudes toward AI and AI self-efficacy in this relationship. Based on the above theoretical considerations, the conceptual framework of the present study is illustrated in Figure 1.

## 2 Methods

### 2.1 Participants

This was a multicenter cross-sectional study conducted between May and October 2025 among nursing students from 11 universities in Sichuan Province, China. The inclusion criteria were age ≥ 18 years and current enrollment in a nursing program. Students who declined to participate or provided illogical responses were excluded. Ethical approval was obtained, and informed consent was collected.

### 2.2 Sample size

The required sample size was estimated using the formula for cross-sectional studies: *n* =(Z_(1-α/2)^∧^2·p·(1-p))/δ2 where the significance level α = 0.05(Z_(1-α/2) = 1.96), the estimated proportion *p* = 0.5 to maximize the sample size, and the allowable error δ = 0.05. Based on this calculation, the theoretical sample size was 385 participants. To account for potential dropout or incomplete responses, an additional 20% was added, resulting in a final target sample size of approximately 481 participants.

### 2.3 Data collection and quality control

This multicenter cross-sectional study employed convenience sampling to recruit nursing students. Data were collected via the online platform SoJump (https://www.wjx.cn/). Participants accessed the questionnaire by scanning a QR code and provided electronic informed consent before proceeding. All responses were anonymized, and each device was restricted to one submission to prevent duplicates. To ensure data quality, participants received standardized instructions on the study objectives and questionnaire completion. Logical consistency checks and double-entry verification were performed to ensure data accuracy.

### 2.4 Measurements

#### 2.4.1 Demographic characteristics

Demographic characteristics were collected using a self-designed questionnaire, including age, gender, ethnicity, family type, education level, academic year, stage of study, and previous experience with artificial intelligence–related training.

#### 2.4.2 Artificial intelligence literacy scale (AILS)

The AILS, developed by Li and Liu (3), is widely used to assess AI literacy. The scale comprises 12 items across four dimensions—awareness, use, evaluation, and ethics—rated on a seven-point Likert scale ranging from 1 (strongly disagree) to 7 (strongly agree). Three items are reverse-coded, and total scores range from 12 to 84, with higher scores indicating greater AI literacy. The AILS has been validated among both nursing students and registered nurses, with Cronbach's α values ranging from 0.829 to 0.85 (24, 34). In the present study, the Cronbach's α coefficient was 0.913.

#### 2.4.3 General attitudes toward artificial intelligence scale (GAAIS)

The GAAIS, developed by Schepman and Rodway (35), comprises 20 items across two subscales—Positive Attitudes (items 1–12) and Negative Attitudes (items 13–20)—rated on a five-point Likert scale from 1 (strongly disagree) to 5 (strongly agree). Items in the negative attitude subscale are reverse-scored when calculating the total score, with total scores ranging from 20 to 100 and higher scores indicating a more positive overall attitude toward AI. The GAAIS has been validated among nursing students in China and demonstrated good internal consistency, with Cronbach's α coefficients of 0.957 and 0.958 for the positive and negative subscales, respectively (36). In the present study, the overall Cronbach's α coefficient for the scale was 0.954, while the coefficients for the positive and negative subscales were 0.950 and 0.948, respectively.

#### 2.4.4 Artificial intelligence self-efficacy scale (AISES)

The AISES, developed by Wang and Chuang (27), comprises 22 items across four dimensions: assistance, anthropomorphic interaction, AI comfort, and technical skills. Each item is rated on a seven-point Likert scale from 1 (strongly disagree) to 7 (strongly agree), with total scores ranging from 22 to 154, where higher scores reflect greater self-efficacy in using AI technologies or products. The scale has been validated among nursing students, demonstrating excellent internal consistency, with a Cronbach's α of 0.982 (36). In the present study, the Cronbach's α coefficient was 0.960.

#### 2.4.5 Artificial intelligence anxiety scale (AIAS)

The AIAS, developed by Wang and Wang (37), comprises 21 items across four subdimensions: Learning, Job Replacement, Sociotechnical Blindness, and AI Configuration. Each item is rated on a seven-point Likert scale from 1 (strongly disagree) to 7 (strongly agree), with total scores ranging from 21 to 147, where higher scores reflect greater levels of AI-related anxiety. The AIAS has been validated among nursing students, demonstrating good internal consistency, with a Cronbach's α of 0.880 (8). In the present study, the Cronbach's α coefficient was 0.948.

### 2.5 Statistical analysis

Data were double-entered and cross-checked using EpiData 3.1 (The EpiData Association, Odense, Denmark) to ensure accuracy. Statistical analyses were performed with SPSS version 26.0 (IBM Corp., Armonk, NY, USA). Harman's single-factor test was conducted on the GAAIS, AILS, AISES, and AIAS to assess potential common method bias.

Descriptive statistics summarized participants' demographics and scale scores. Categorical variables were reported as frequencies and percentages, and continuous variables were tested for normality using the Shapiro–Wilk test. Normally distributed data were expressed as mean ± standard deviation (x ± s), and non-normally distributed data as median and interquartile range [M (Q1, Q3)]. Group comparisons used independent-samples *t*-tests or one-way ANOVA with post hoc tests for normally distributed data with equal variances, and Mann–Whitney U or other nonparametric tests when assumptions were violated. Pearson's correlation analysis examined associations among GAAIS, AILS, AISES, and AIAS scores. Structural equation modeling (SEM) was performed using AMOS version 26.0 to examine the hypothesized associations among the four study variables. Dimension-level composite scores were used as indicators of the four constructs rather than modeling all individual items. This approach was chosen to keep the measurement model parsimonious and avoid an excessively complex item-level model while retaining the established multidimensional structure of each scale. The high internal consistency observed for all scales supported this approach; however, the use of dimension-level composites may obscure item-level variation. All tests were two-tailed, with *P* < 0.05 considered statistically significant.

### 2.6 Ethics approval

This study was conducted in accordance with the ethical principles of the Declaration of Helsinki (2,000 revision). Ethical approval was obtained from the Ethics Committee of West China Second University Hospital, Sichuan University (Approval No: 2025–050). Written informed consent was obtained from all participants prior to data collection.

## 3 Results

### 3.1 Participant sociodemographic characteristics

Of 1,573 surveys, 1,482 were valid (94.2%). Questionnaires were excluded due to refusal to participate (*n* = 6, 0.4%), completion time under 120 seconds (*n* = 64, 4.1%), or inconsistent responses to reverse-coded items (*n* = 21, 1.3%).

Table 1 presents the sociodemographic characteristics of the participants. Of the 1,482 nursing students, 88.5% were female (*n* = 1,324) and 11.5% male (*n* = 158), with a mean age of 20.3 ± 1.7 years. The majority were of Han nationality (86.2%, *n* = 1,278) and from nuclear families (84.6%, *n* = 1,254).

**Table 1:** Differences in AI attitudes, AI literacy, AI self-efficacy and AI anxiety across general characteristics of participants (*n* = 1482).

Variable		*N*(%)	Total score of AI general attitudes			Total score of AI literacy			Total score of AI self-efficacy			Total score of AI anxiety		
			*M ±SD*	*t/F*	*P*	*M ±SD*	*t/F*	*P*	*M ±SD*	*t/F*	*P*	*M ±SD*	*t/F*	*P*
		68.82 ± 16.41			52.55 ± 13.51			98.81 ± 25.98			76.45 ± 23.31		
Gender	Male	158 (10.66)	66.40 ± 17.85	1.668	0.095	52.20 ± 13.85	−0.364	0.730	98.22 ± 27.02	−0.302	0.763	77.13 ± 25.97	0.038	0.701
	Female	1324 (89.34)	69.11 ± 16.21			52.60 ± 13.48			98.99 ± 25.86			76.37 ± 22.98		
Age (years)		20.28 ± 1.67												
Nationality	Han	1278 (86.23)	68.28 ± 16.61	2.922	0.003	52.46 ± 13.46	−0.681	0.496	98.28 ± 26.10	−1.954	0.051	76.62 ± 23.16	0.701	0.480
	Others	204 (13.77)	72.24 ± 14.63			53.15 ± 13.90			102.11 ± 25.01			75.38 ± 24.23		
Family structure	Intact (original) family	1254 (84.62)	68.68 ± 16.36	0.335	0.715	52.40 ± 13.53	0.984	0.374	98.61 ± 25.90	0.504	0.604	76.48 ± 23.146	0.523	0.593
	Single-parent family	167 (11.27)	69.77 ± 16.67			53.92 ± 13.81			100.66 ± 26.30			75.37 ± 24.83		
	Reconstituted (blended) family	61 (4.11)	69.15 ± 16.75			51.98 ± 26.88			97.80 ± 26.88			78.92 ± 22.50		
Educational level	Junior college	819 (55.26)	69.52 ± 15.87	1.763	0.172	52.56 ± 13.29	0.234	0.791	100.20 ± 25.81	2.662	0.070	78.18 ± 23.503	5.084	0.006
	Bachelor's degree	640 (43.19)	67.90 ± 17.08			52.62 ± 13.84			97.04 ± 26.01			74.04 ± 28.96		
	Postgraduate degree	23 (1.55)	69.35 ± 15.44			50.65 ± 12.80			98.48 ± 29.08			73.04 ± 28.96		
Academic year	Year 1	214 (14.44)	73.80 ± 14.04	6.738	0.01	51.53 ± 12.49	0.811	0.518	100.31 ± 24.79	0.461	0.764	77.93 ± 24.70	1.092	0.359
	Year 2	238 (16.06)	69.58 ± 15.98			53.54 ± 12.19			99.30 ± 26.08			78.31 ± 24.70		
	Year 3	817 (55.13)	67.80 ± 16.79			52.39 ± 13.62			98.05 ± 26.08			75.67 ± 23.29		
	Year 4	183 (12.35)	66.67 ± 16.79			53.31 ± 14.28			99.92 ± 26.48			75.27 ± 23.49		
	Year 5	30 (2.02)	68.20 ± 16.15			51.87 ± 15.40			98.30 ± 25.02			79.72 ± 22.13		
Learning stage	Theoretical coursework	831 (56.07)	69.41 ± 16.241	1.561	0.119	52.76 ± 13.46	0.653	0.514	98.50 ± 26.01	−0.518	0.605	76.00 ± 23.25	−0.846	0.398
	Clinical internship	651 (43.93)	68.07 ± 16.60			52.29 ± 13.58			99.21 ± 25.96			77.03 ± 23.39		
AI-related training experience	Formal training	247 (16.67)	69.15 ± 15.79	0.552	0.576	53.96 ± 13.46	3.263	0.039	103.34 ± 26.67	4.619	0.010	74.94 ± 25.24	0.922	0.398
	Self-learning	622 (41.97)	68.30 ± 17.51			52.97 ± 13.89			98.23± 26.56			76.25 ± 24.11		
	No training experience	613 (41.36)	69.22 ± 15.47			51.57 ± 13.09			97.57 ± 24.93			77.27 ± 21.61		

Exposure to AI-related education was limited. Only 16.7% (*n* = 247) had received formal training, 42.0% (*n* = 622) had acquired knowledge through self-directed learning, and 41.4% (*n* = 613) reported no prior exposure. These findings suggest that overall coverage of AI education among nursing students remains low, with formal training still at an early stage.

### 3.2 AI literacy, AI attitudes, AI self-efficacy, AI anxiety, and associated factors

The mean scores for AI literacy, AI attitudes, AI self-efficacy, and AI anxiety were 52.55 ± 13.51, 68.82 ± 16.41, 98.81 ± 25.98, and 76.45 ± 23.31, respectively. Analysis by demographic characteristics revealed significant differences in several of the scales (Table 1).

### 3.3 Correlations among AI literacy, AI attitudes, AI self-efficacy, and AI anxiety

Pearson correlation analysis was conducted to examine the relationships among AI literacy, AI attitudes, AI self-efficacy, and AI anxiety, with results presented in Table 2. AI literacy was positively correlated with AI attitudes (*r* = 0.285, *P* < 0.01) and AI self-efficacy (*r* = 0.509, *P* < 0.01), and negatively correlated with AI anxiety (*r* = −0.434, *P* < 0.01). AI attitudes were positively associated with AI self-efficacy (*r* = 0.359, *P* < 0.01) and negatively associated with AI anxiety (*r* = −0.314, *P* < 0.01). Furthermore, AI self-efficacy was negatively correlated with AI anxiety (*r* = −0.425, *P* < 0.01).

**Table 2:** Correlation analysis among AI literacy, AI attitudes, AI self-efficacy, and AI anxiety (*n* = 1482).

Variable	AI literacy	AI attitudes	AI self-efficacy	AI anxiety
AI literacy	1			
AI attitudes	0.285^**^	1		
AI self-efficacy	0.509^**^	0.359^**^	1	
AI anxiety	−0.434^**^	−0.314^**^	−0.425^**^	1

^**^P < 0.01.

### 3.4 Mediation effect analysis of AI attitudes, literacy, AI self-efficacy, and AI anxiety

**Figure 2:**
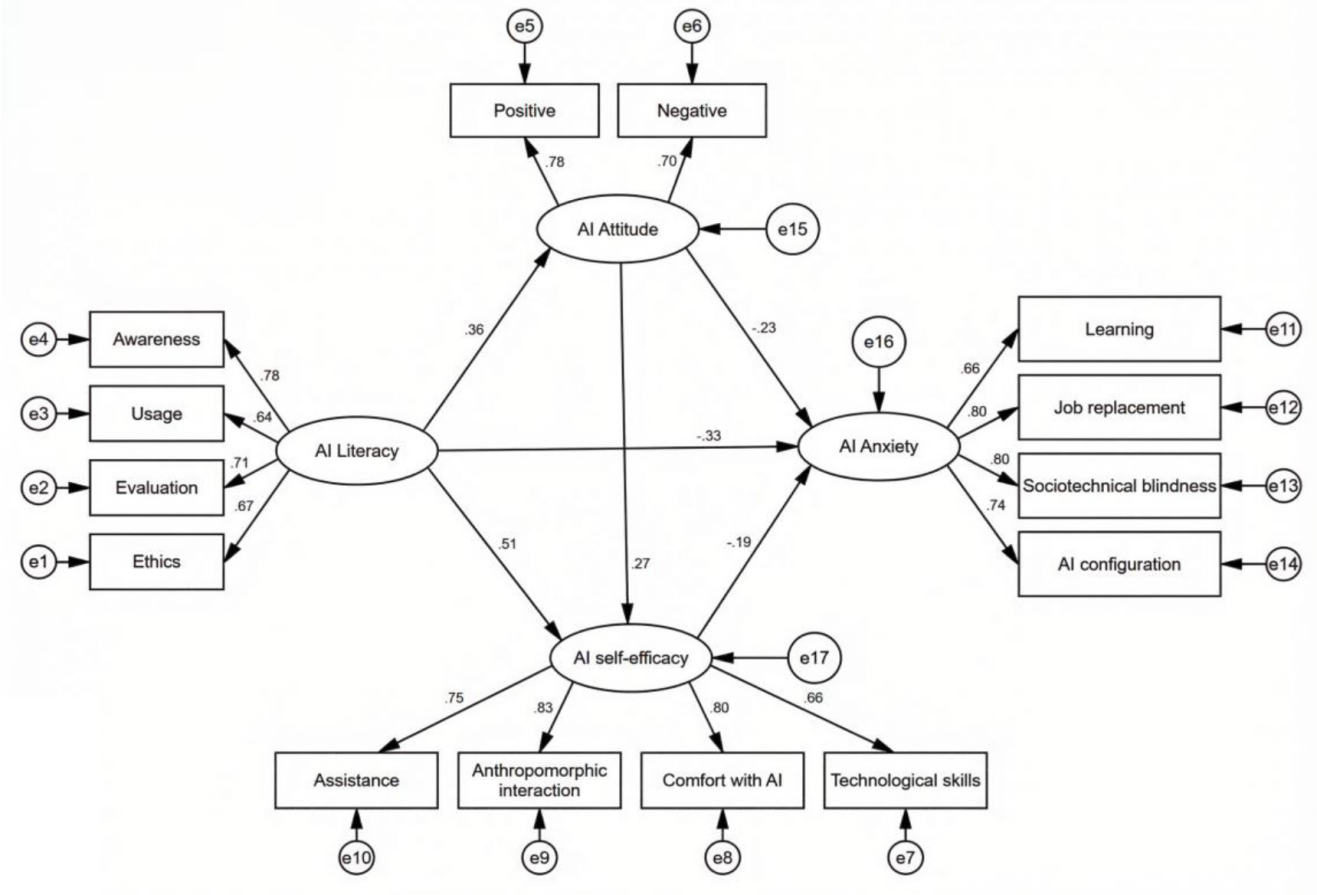
Mediation effect analysis of AI general attitudes, AI literacy, AI self-efficacy, and AI anxiety.

SEM was constructed using AMOS 26.0 to examine the mediating effects of AI attitudes and AI self-efficacy on the relationship between AI literacy and AI anxiety. The model demonstrated acceptable fit (χ^2^/df = 4.516, RMSEA = 0.049, TLI = 0.958, CFI = 0.968, SRMR < 0.049), indicating that the hypothesized relationships among variables were well represented (Figure 2).

Path analysis (Table 3) revealed that AI literacy had significant positive effects on AI attitudes (β = 0.365, *P* < 0.001) and AI self-efficacy (β = 0.512, *P* < 0.001). AI attitudes were positively associated with AI self-efficacy (β = 0.269, *P* < 0.001). AI literacy also exerted a significant negative direct effect on AI anxiety (β = −0.329, *P* < 0.001). Additionally, AI attitudes (β = −0.234, *P* < 0.001) and AI self-efficacy (β = −0.191, *P* < 0.001) had significant negative effects on AI anxiety, indicating that AI literacy indirectly influenced AI anxiety through both AI attitudes and self-efficacy.

**Table 3:** Mediation effect analysis of AI attitudes, AI literacy, AI self-efficacy, and AI anxiety (*n* = 1482).

Path	Estimate	*SE*	*t*	*P*
AI attitudes ← AI literacy	0.365	0.028	10.154	^***^
AI self-efficacy ← AI literacy	0.512	0.039	13.924	^***^
AI self-efficacy ← AI attitudes	0.269	0.046	7.867	^***^
AI anxiety ← AI literacy	−0.329	0.041	−7.990	^***^
AI anxiety ← AI attitudes	−0.234	0.048	−6.282	^***^
AI anxiety ← AI self-efficacy	−0.191	0.039	−4.673	^***^

Using AMOS with 5,000 bootstrap resamples, the mediating effects of AI literacy on AI anxiety were examined (Table 4). AI literacy demonstrated a significant total effect on AI anxiety (β = −0.531, SE = 0.030, 95% CI: −0.586 to −0.468) and a significant direct effect (β = −0.329, SE = 0.041, 95% CI: −0.409 to −0.248), indicating a robust direct negative association.

**Table 4:** Decomposition table of mediation effects for AI literacy, AI attitudes, AI self-efficacy, and AI anxiety (*n* = 1482).

Mediating effect pathways		Estimate	*SE*	95%*CI*		*P*
				Lower	Upper	
Total effect	AILS → AIAS	−0.531	0.030	−0.586	−0.468	^***^
Direct effect	AILS → AIAS	−0.329	0.041	−0.409	−0.248	^***^
Indirect effects	AILS → GAAIS → AIAS	−0.085	0.014	−0.117	−0.059	^***^
	AILS → AISES → AIAS	−0.098	0.022	−0.143	−0.055	^***^
	AILS → GAAIS → AISES → AIAS	−0.019	0.005	−0.031	−0.010	^***^

^***^P < 0.001.

Indirect effect analyses showed that the AI literacy → AI attitudes → AI anxiety pathway yielded an effect of −0.085 (SE = 0.014, 95% CI: −0.117 to −0.059), and the AI literacy → AI self-efficacy → AI anxiety pathway yielded an effect of −0.098 (SE = 0.022, 95% CI: −0.143 to −0.055). The chain mediation via AI attitudes and AI self-efficacy (AI literacy → AI attitudes → AI self-efficacy → AI anxiety) produced an effect of −0.019 (SE = 0.005, 95% CI: −0.031 to −0.010). All indirect effects' 95% confidence intervals excluded zero, indicating that AI attitudes and AI self-efficacy each partially mediate the relationship between AI literacy and AI anxiety, and that a significant serial mediation also exists.

## 4 Discussion

### 4.1 Current status of nursing students' AI literacy, AI attitudes, AI self-efficacy, and AI anxiety

The findings indicate that nursing students' AI anxiety scores averaged 76.45 ± 23.31, representing a moderate level, which aligns with previous research (6, 8). Prior studies have shown that most nursing students possess limited knowledge of AI, with only 44% demonstrating a moderate or higher understanding of its principles and applications (38). Insufficient technological awareness often increases uncertainty regarding new technologies, which can trigger anxiety (8).

In this study, AI literacy scores averaged 52.55 ± 13.51, indicating a moderate level, consistent with findings by Akca Sumengen et al. (13) and Boztepe et al. (24). In Chinese higher education institutions, AI applications remain largely confined to classroom, course, and student management, with limited integration into professional nursing curricula (39). Although some medical schools have begun offering AI-related courses, curriculum structures, teaching objectives, and training models remain exploratory and lack standardized guidance (40). Consequently, there remains substantial potential for improving nursing students' AI literacy.

Students' mean AI attitudes score was 68.82 ± 16.41, reflecting generally positive attitudes, in line with prior studies (13, 20, 24). With the widespread adoption of generative AI tools, students increasingly use AI for grammar checking, assignment assistance, literature searches, and research idea generation (41). Direct exposure to these practical benefits may foster more positive attitudes toward AI.

The mean AI self-efficacy score was 98.81 ± 25.98, indicating a moderate level, consistent with the findings of He et al. (15). Previous evidence suggests that students' AI self-efficacy closely relates to their preparedness for AI use (42). In this study, only 16.67% of students had formal AI training, 41.97% were self-taught, and 41.36% had no prior AI learning experience, indicating relatively limited preparation and potentially constraining the development of AI self-efficacy.

### 4.2 AI literacy and AI anxiety

This study found a significant negative correlation between AI literacy and AI anxiety, supporting Hypothesis 1. Thus, students with higher AI literacy tended to report lower AI anxiety, consistent with Schiavo et al. (18). Because the data were cross-sectional, this association should not be interpreted as evidence that improving AI literacy will necessarily reduce anxiety. Previous research indicates a positive relationship between AI literacy and AI readiness, suggesting that more literate students may be better prepared and more open to adopting and using AI technologies (24).

Higher AI readiness may be associated with less uncertainty when encountering new technologies and, consequently, lower anxiety (43). Students with higher AI literacy may also have stronger understanding and application skills, together with greater confidence and adaptability when facing AI-related challenges (28). Greater confidence in using AI has been associated with lower anxiety during interactions (32). The present study also observed that students who had received AI training reported higher AI literacy, consistent with prior research (14, 44); however, the direction of this relationship cannot be determined from the present design.

These findings have practical implications for nursing education, although intervention studies are needed before effectiveness can be inferred. AI literacy can be embedded across foundational, professional, and clinical nursing courses rather than confined to a single elective. Simulation-based activities may allow students to practice using AI-supported decision tools in realistic scenarios, evaluate outputs, identify errors, and reflect on human oversight. Supervised exposure to appropriate clinical AI tools during practice placements may help connect classroom knowledge with clinical application. Curricula should also include AI ethics, privacy, bias, accountability, and patient communication. Faculty development programs are needed to strengthen educators' AI knowledge, teaching skills, and ability to guide safe and critical use of AI. Structured workshops and staged skills training may additionally provide students with repeated opportunities to build familiarity and confidence (38, 45, 46).

### 4.3 Mediating role of AI attitudes

The results showed a significant indirect association through AI attitudes, supporting Hypothesis 2. Students with higher AI literacy may be better able to critically evaluate AI's advantages and limitations and maintain a balanced perspective toward exaggerated positive or negative portrayals (14). Such understanding may be associated with more favorable AI attitudes.

More favorable attitudes may be related to greater confidence when interacting with digital technologies and to lower anxiety during AI use (27). Attitudes are associated not only with perceived usefulness and ease of use but also with awareness of potential benefits and risks and with the surrounding social and educational environment (47). Recognizing AI's practical value—such as improved efficiency, support for clinical decision-making, and optimized nursing workflows—may contribute to more favorable attitudes (38). Thus, nursing programs should cultivate AI competencies while creating supportive environments that allow students to experience AI's practical benefits. Case-based teaching, scenario simulations, or clinical demonstrations may help students understand AI's role in improving nursing quality and support balanced attitudes (38). Clear legal frameworks, accountability mechanisms, privacy protections, informed consent prompts, privacy dashboards, and accessible data policies may also strengthen transparency and trust in AI (46).

### 4.4 Mediating role of AI self-efficacy

AI self-efficacy showed a significant indirect association between AI literacy and AI anxiety, supporting Hypothesis 3. Students with higher AI literacy may possess stronger technical understanding and operational skills, enabling them to analyze complex data, participate in interdisciplinary collaboration, and explore innovative AI applications in nursing (48). These experiences may be associated with greater confidence in using AI.

Higher AI self-efficacy reflects belief in one's ability to use AI effectively and maintain control during interactions (30, 49) and may therefore be related to lower uncertainty and technology-related anxiety. Prior studies suggest that mastery of technology is closely related to self-efficacy (42). Systematic training and hands-on opportunities may help strengthen nursing students' AI self-efficacy and may be associated with lower anxiety; however, intervention research is needed to test these effects.

### 4.5 Sequential mediation of AI attitudes and AI self-efficacy

The serial mediation model indicated that AI literacy was associated with AI anxiety not only through AI attitudes or self-efficacy separately but also through the proposed sequence “AI attitudes → AI self-efficacy,” supporting Hypothesis 4. In this ordering, AI attitudes are conceptualized as the earlier evaluative response: students first interpret AI as useful, acceptable, threatening, or risky on the basis of their knowledge and beliefs. A more favorable evaluation may be associated with greater willingness to engage with AI tools. Engagement may provide practice and mastery experiences, which are closely related to self-efficacy. Self-efficacy is more proximal to anxiety because confidence and perceived control may shape whether AI-related tasks are experienced as manageable or threatening. The observed positive association between AI attitudes and self-efficacy is consistent with prior findings (32, 50, 51). However, because all variables were measured at one time point, the statistical ordering does not establish temporal precedence, and alternative or reciprocal pathways remain possible.

Overall, all proposed hypotheses were supported at the level of statistical association. Higher AI literacy was associated with lower AI anxiety directly and through the proposed indirect pathways involving attitudes and self-efficacy; these findings should not be interpreted as causal.

### 4.6 Strengths

This study has several strengths. First, it employed a multicenter survey design and included 1,482 nursing students from 11 universities, providing substantial statistical power and coverage across multiple institutions within Sichuan Province. However, the multicenter design should not be taken to imply national or international representativeness. Second, the study systematically examined the associations linking AI literacy and AI anxiety, including separate and serial indirect pathways. Structural equation modeling and bootstrap analysis provided insight into the proposed pattern of relationships among AI literacy, attitudes, self-efficacy, and anxiety. Finally, multiple validated instruments were employed (AILS, GAAIS, AISES, and AIAS), all demonstrating high internal consistency and supporting measurement reliability.

### 4.7 Limitations

Despite its strengths, the study has several limitations. First, the cross-sectional design does not establish temporal sequence or causality; the mediation paths should therefore be interpreted as statistical associations, and reverse or reciprocal relationships remain possible. Second, all variables were self-reported in the same survey. Although Harman's single-factor test was conducted, this diagnostic alone cannot rule out common method variance, and social desirability, recall, or response-style biases may remain. Third, convenience sampling from 11 universities within a single Chinese province limits external validity. Nursing curricula, access to AI, institutional support, cultural attitudes, and prior AI exposure may differ across regions and countries; the findings should therefore not be generalized to all nursing students or educational systems. Fourth, the model focused on AI literacy, attitudes, and self-efficacy and did not include other potential determinants of AI anxiety, such as digital literacy, technology readiness, trust in AI, organizational support, direct experience with AI, personality, learning context, and cultural factors. Finally, using dimension-level composite indicators improved model parsimony but may have obscured item-level variation.

### 4.8 Future directions

Future studies should use longitudinal designs to examine temporal ordering and possible reciprocal relationships among AI literacy, attitudes, self-efficacy, and anxiety. Randomized or quasi-experimental studies could test whether structured AI education changes these variables over time. Multiregional and multinational studies using more representative sampling strategies would help assess differences across cultural and educational contexts. Research should also include practicing nurses and other healthcare professionals and examine contextual factors such as organizational support, technology readiness, trust in AI, and direct clinical experience with AI tools.

## 5 Conclusion

Nursing students reported moderate AI literacy, self-efficacy, and anxiety and generally positive attitudes toward AI. Higher AI literacy was associated with more favorable attitudes, stronger self-efficacy, and lower AI anxiety. The SEM also supported indirect associations through attitudes and self-efficacy, including the proposed serial pathway; however, these cross-sectional findings do not establish causality or temporal ordering. Nursing programs may consider integrating AI literacy, simulation-based practice, supervised use of clinical AI tools, ethics education, and faculty development, while future longitudinal and intervention studies are needed to determine whether such strategies reduce AI anxiety.

## Data Availability

The raw data supporting the conclusions of this article will be made available by the authors, without undue reservation.
